# A Few Cases of Replantation

**Published:** 1901-05

**Authors:** R. W. Hunter

**Affiliations:** Greenfield, Mass.


### A FEW CASES OF REPLANTATION.[1]

BY DR. R. W. HUNTER, GREENFIELD, MASS.

Before reading you the history of these few cases, I wish to state that I look on it as a last resort and allowable only as such. Nearly all of the cases in which I have resorted to replantation have been the result of alveolar abscess which I found incurable by other methods.

[1] Read before The New York Institute of Stomatology, February 5, 1901.

My first case of replantation was in June, 1895, a superior left bicuspid. The tooth had been under treatment for an abscess for many weeks, but a sharp curve at the end of the root baffled me, and I resorted to replantation. The tooth was extracted while the patient was under the influence of an anæsthetic. The curved portion of the root was cut away, the root and canals cleansed with listerine and warm water, the canals filled with gutta-percha, and the cavity in the tooth filled with amalgam. Then the socket was very carefully cleansed by syringing with listerine and warm water. The tooth was then replaced in the socket, which required some force. The tooth was quite sore for about ten days, after which the pain and swelling gradually disappeared.

In July, 1897, the same patient presented herself with the superior first bicuspid on the right side in much the same condition. This tooth had been previously treated and the canals filled. In my endeavor to remove the filling from the canals the root was perforated. The tooth was extracted and the same course pursued as with the other tooth, with equally good results.

Both of these teeth are at present firmly fixed in the alveolus and doing good service. The patient is a young lady of twenty-two or twenty-four years of age.

The next case came on July 22, 1897, the patient being a woman of about forty years of age. The tooth was a lower first bicuspid. Opening the canals, I discovered that they had been filled with cotton. Further investigation proved that the wall of the canal had been perforated. Not being satisfied with one perforation, I attempted to open into the canal beyond perforation, and succeeded in penetrating the wall myself. Replantation was resorted to, and within two weeks the tooth was comparatively firm. I had the pleasure of examining it last fall, and found it perfectly firm.

The next case is one which proved a failure. Happily, I can say the work was not mine, although the same result would probably have followed. The assistant in the office did it for one of his relatives. The operation appeared successful. Within two years the crown broke off and a Richmond crown was affixed. Within the next two years absorption set in, and the root was easily removed with an excavator.

The next case I shall tell you of is the most unfavorable of any I have ever attempted. The patient had lost the superior twelfthyear molar early in life, and the third molar had moved forward to fill the space, tipping forward slightly. On opening into it to relieve an abscess, I found the canals filled with cement. Finding I could not get them clear without perforating the root, I removed the tooth and filled the tips of the roots and the cavity I had made, and replaced the tooth. Other cases had been easily retained by a combination of rubber dam and silk ligature; but for this I devised a cradle of annealed brass wire. This operation was performed October 27, 1899, and at present the tooth, although not perfectly firm, is better than before.

The last case I have to tell you of is more of a credit to Dame Nature than myself. On October 4, 1900, I replanted a superior right first molar. The next day the patient was taken with typhoid fever. When I heard of that I made up my mind that a failure would surely result. About a month ago the patient came in, and, to my surprise, the result is perfect.

